# Preparation and Characterization of *Ocimum basilicum* Extract‐Loaded Niosomes and Evaluation of Their Cytotoxic and Pro‐Apoptotic Potential in SKOV3 Ovarian Cancer Cells

**DOI:** 10.1002/cbdv.71779

**Published:** 2026-09-29

**Authors:** Zahra Mousavi, Mahsa Kavousi, Mahnaz Farahmand

**Affiliations:** ^1^ Department of Biology, ET.C. Islamic Azad University Tehran Iran

**Keywords:** modulation of metastasis‐associated gene expression, niosome, *Ocimum basilicum* L., pro‐apoptotic, SKOV3 cell line

## Abstract

Niosomes are promising nanocarriers for improving the stability, bioavailability, and delivery of plant‐derived bioactive compounds. This study aimed to prepare and physicochemically characterize niosomes loaded with *Ocimum basilicum* L. extract and evaluate their anticancer and metastasis‐related effects in human ovarian cancer SKOV3 cells. The niosomes were characterized using DLS, SEM, FTIR, and in vitro release analysis, showing a mean particle size of 256.2 ± 0.21 nm, PDI of 0.265 ± 0.007, zeta potential of −35.4 ± 0.3 mV, and encapsulation efficiency of 85.2 ± 0.6%. Cytotoxicity was assessed by MTT assay after 24, 48, and 72 h, and the 72 h IC_50_ (398.24 µg/mL) was used for subsequent analyses. The extract‐loaded niosomes exhibited significantly greater cytotoxicity than the free extract and empty niosomes, particularly after 72 h. qRT‐PCR revealed significant upregulation of Bax (3.46‐fold) and downregulation of Bcl2 (0.67‐fold), MMP2 (0.63‐fold), and MMP9 (0.64‐fold) (*p* < 0.001). Flow cytometry confirmed enhanced apoptosis, with 27.8% apoptotic and 5.87% necrotic cells. Overall, *O. basilicum* extract‐loaded niosomes increased apoptosis and modulated apoptosis‐ and metastasis‐associated genes, supporting their potential as nanocarriers for plant‐derived anticancer compounds.

## Graphical abstract

*Ocimum basilicum* extract was successfully encapsulated in niosomal nanocarriers, resulting in a sustained‐release formulation with favorable physicochemical characteristics. In SKOV3 ovarian cancer cells, the extract‐loaded niosomes showed enhanced cytotoxicity compared with the free extract and blank niosomes. The treatment increased apoptosis, accompanied by upregulation of the pro‐apoptotic gene Bax and downregulation of Bcl2, MMP2, and MMP9. These findings indicate that *O. basilicum* extract‐loaded niosomes possess promising cytotoxic and pro‐apoptotic potential in vitro and may serve as a potential nanocarrier for plant‐derived anticancer compounds.

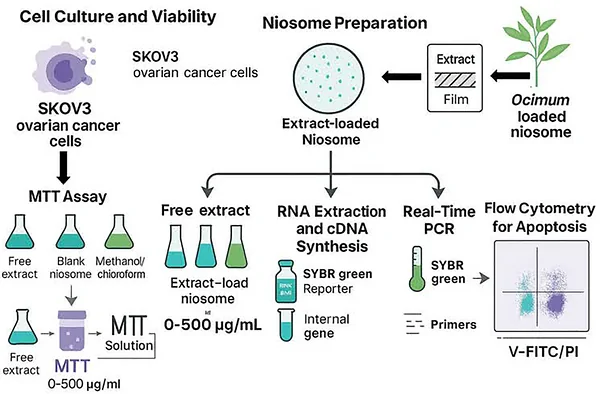

## 1 Introduction

Ovarian cancer is one of the most aggressive gynecological malignancies worldwide and remains among the leading causes of cancer‐related mortality in women, with approximately 300 000 new cases and more than 200 000 deaths reported annually [1]. In Iran, ovarian cancer represents a significant proportion of female malignancies, and its incidence has gradually increased over recent years [2]. Unfortunately, the absence of effective early screening strategies and the frequent diagnosis at advanced stages contribute to its poor prognosis and high mortality rate [3]. The disease is particularly challenging because of its high metastatic potential and the development of resistance to conventional therapeutic approaches, emphasizing the urgent need for more effective treatment strategies [4]. Additionally, cancers of the female reproductive system, including ovarian and breast cancers, are frequently associated with infertility, either as a direct consequence of the disease or as a result of anticancer treatments, highlighting their broader reproductive and clinical implications [5, 6, 7]. Furthermore, ovarian and breast cancers share several molecular characteristics, including dysregulation of estrogen signaling, the PI3K/AKT pathway, and p53 signaling, which contribute to tumor progression, metastasis, and therapeutic resistance [8, 9].

*Ocimum basilicum*, commonly known as sweet basil, is an aromatic herb belonging to the family Lamiaceae. It is generally cultivated as an annual plant and is characterized by erect, branched stems, ovate leaves, and terminal inflorescences bearing white to pink flowers. The species is native to tropical and subtropical regions of Asia and is now widely cultivated worldwide [10].

The leaves and aerial parts of *O. basilicum* contain a diverse range of bioactive compounds, including polyphenols, phenolic acids, flavonoids, and volatile constituents. The composition and abundance of these compounds can vary considerably depending on the cultivar and growing conditions. These phytochemicals have been associated with various biological activities, including antioxidant, anti‐inflammatory, antimicrobial, and potential anticancer effects [10, 11]. Its biological activities are mainly attributed to its diverse phytochemical composition, including compounds that may modulate cellular processes involved in cancer progression [10]. These bioactive compounds exhibit antioxidant, anti‐inflammatory, antimicrobial, and anticancer activities, making basil an attractive source of naturally derived therapeutic agents [12]. At the molecular level, basil‐derived compounds have been reported to regulate apoptosis by increasing the expression of pro‐apoptotic mediators, including *Bax* and caspases, while suppressing anti‐apoptotic proteins such as Bcl2. They have also been shown to inhibit the expression of metastasis‐associated genes, including *MMP2*, *MMP9*, and *VEGF*, which play essential roles in extracellular matrix degradation, tumor invasion, and angiogenesis [13, 14]. Similar molecular alterations have been described in breast cancer, where impaired apoptosis and enhanced metastatic signaling contribute to tumor progression and therapeutic resistance [15, 16]. Collectively, these findings suggest that basil‐derived phytochemicals may modulate multiple molecular pathways involved in cancer progression, supporting their potential application in cancer therapy [17].

Advances in nanotechnology have provided new opportunities to improve the therapeutic efficacy of plant‐derived bioactive compounds by enhancing their stability, bioavailability, and intracellular delivery [18]. Niosomes, non‐ionic surfactant‐based vesicular nanocarriers, have attracted considerable attention because of their biocompatibility, chemical stability, controlled drug‐release properties, and ability to improve cellular uptake while potentially reducing systemic toxicity [19]. Previous studies have demonstrated that encapsulating plant extracts or phytochemicals in niosomal formulations enhances their biological activities, including increased cytotoxicity toward cancer cells, improved induction of apoptosis, and suppression of metastasis‐related pathways, compared with the corresponding free compounds [20, 21, 22].

Despite these promising findings, the anticancer potential of *O. basilicum* extract encapsulated in niosomal nanocarriers has not been comprehensively investigated in SKOV3 ovarian cancer cells. Therefore, the novelty of the present study lies primarily in the development and physicochemical characterization of a specific *O. basilicum* extract‐loaded niosomal formulation and its integrated in vitro evaluation in SKOV3 ovarian cancer cells, combining cytotoxicity assessment, flow‐cytometric analysis of apoptosis, and characterization of associated transcriptional responses. Accordingly, this study aimed to investigate the cytotoxic and pro‐apoptotic potential of *O. basilicum* extract‐loaded niosomes of *O. basilicum* extract‐loaded niosomes as a plant‐based nanocarrier system, thereby providing further evidence for their potential application in ovarian cancer research.

## 2 Materials and Methods

### 2.1 Nanoniosome Preparation and Physicochemical Characterization of Niosomes

Niosomes were prepared using the thin‐film hydration method. Cholesterol, Span 60, and *O. basilicum* extract were dissolved in a chloroform:methanol (2:1, v/v) mixture to obtain a homogeneous lipid phase. The organic solvent was removed under reduced pressure using a rotary evaporator to form a thin lipid film on the inner wall of a round‐bottom flask. The dried lipid film was hydrated with phosphate‐buffered saline (PBS, pH 7.2) at 60°C for 30 min, followed by 7 min of probe sonication to reduce vesicle size and obtain a homogeneous nanosuspension [21].

#### 2.1.1 Particle Size, Polydispersity Index (PDI), and Zeta Potential

Particle size distribution, polydispersity index (PDI), and zeta potential of the prepared niosomes were determined by dynamic light scattering (DLS) using a Malvern Zetasizer (Malvern Instruments Ltd., UK). Prior to analysis, the niosomal suspension was diluted 1:100 with distilled water, and all measurements were performed at a 90° scattering angle at room temperature [21].

#### 2.1.2 Scanning Electron Microscopy (SEM)

The morphology of the prepared niosomes was examined using a Hitachi S‐3400N scanning electron microscope (SEM, Japan). Briefly, 15 µL of the niosomal suspension was placed onto a clean glass slide and allowed to air‐dry at room temperature. The dried samples were mounted on aluminum stubs, sputter‐coated with a thin layer of gold to improve conductivity, and observed at an accelerating voltage of 20 kV [21].

#### 2.1.3 Fourier Transform Infrared (FTIR) Spectroscopy

Fourier‐transform infrared spectroscopy (FTIR) was performed using a Spectrum Two FTIR spectrometer (PerkinElmer, USA) to investigate possible interactions between *O. basilicum* extract and the niosomal components. Samples were compressed using a hydraulic press for 5 min, and spectra were recorded over the range of 400–4000 cm^−^^1^ [21].

#### 2.1.4 Encapsulation Efficiency

The encapsulation efficiency (EE%) of the prepared niosomes was determined by separating the non‐encapsulated extract from the niosomal suspension using the method described previously. The encapsulation efficiency was calculated according to the following equation [23]:

$$\begin{array}{ccc} & & \text{Encapsulation Efficiency}(\%)=[(\text{Drug added}\;-\\ & & \;\text{Free “unentrapped drug”})/\text{Drug added}]\times 100 \end{array}$$

#### 2.1.5 In Vitro Drug Release Study

The in vitro release profile of *O.basilicum* extract‐loaded niosomes was evaluated using the dialysis bag diffusion method. Briefly, an appropriate volume of the niosomal suspension was transferred into a dialysis membrane and immersed in phosphate‐buffered saline under continuous stirring at 37°C. At predetermined time intervals, aliquots of the release medium were withdrawn and replaced with an equal volume of fresh buffer to maintain sink conditions. The amount of released extract was determined spectrophotometrically, and the cumulative percentage of drug release was calculated [23].

### 2.2 Cell Culture and Viability

The human ovarian cancer cell line SKOV3 (IBRC Cat. No. C1000858; RRID: CVCL_0532) was obtained from the Iranian Biological Resource Center (IBRC, Tehran, Iran) and cultured in RPMI‐1640 medium (Gibco, USA) supplemented with 10% (v/v) fetal bovine serum (FBS; Gibco, USA), 100 U/mL penicillin, and 100 µg/mL streptomycin (Gibco, USA) at 37°C in a humidified atmosphere containing 5% CO_2_. Cells were maintained until reaching 70%–80% confluence, with the culture medium replaced every 2–3 days. Cells were detached using 0.25% trypsin–EDTA, centrifuged, and resuspended in fresh culture medium. Cell viability was determined by the trypan blue exclusion assay, and only cultures exhibiting **>**90% viability were used for subsequent experiments.

For the cytotoxicity assay, 1 × 10^4^ cells per well were seeded into 96‐well plates and allowed to attach overnight. Cells were then treated with different concentrations (5.62–500 µg/mL) of free *O. basilicum* extract, blank niosomes, or extract‐loaded niosomes for 24, 48, and 72 h. After each incubation period, 100 µL of MTT solution (0.5 mg/mL in PBS) was added to each well and incubated for 3 h at 37°C. The medium was carefully removed, and the resulting formazan crystals were dissolved in 100 µL dimethyl sulfoxide (DMSO). Absorbance was measured at 570 nm using an ELISA microplate reader (ChroMate, Awareness Technology Inc., USA). Cell viability was calculated using the following equation:

$$\mathrm{Cell}\;\mathrm{viability}\;\left(\%\right)=\frac{\mathrm{ODT}}{\mathrm{ODC}}\;\times 100$$

where ODT represents the optical density of treated cells and ODC represents the optical density of untreated control cells. Dose–response curves were generated, and IC_50_ values were calculated using Pharm‐PCS software. The IC_50_ value obtained after 72 h of treatment was subsequently used for flow cytometry and gene expression analyses [23].

### 2.3 RNA Extraction, cDNA Synthesis, and Real‐Time PCR Analysis

Following treatment with the 72 h IC_50_ concentration of *O. basilicum* extract‐loaded niosomes, total RNA was extracted from SKOV3 cells using RNX‐Plus reagent (Sinagen, Tehran, Iran) according to the manufacturer's instructions. The concentration and purity of the extracted RNA were evaluated spectrophotometrically before complementary DNA (cDNA) synthesis. First‐strand cDNA was synthesized using the RevertAid First Strand cDNA Synthesis Kit (Thermo Scientific/Fermentas, Lithuania) according to the manufacturer's protocol.

Quantitative real‐time PCR was performed using an Aus Diagnostics Real‐Time PCR System (Australia) and SYBR Green PCR Master Mix (Amplicon, Denmark). The expression levels of the apoptosis‐related genes (*Bax* and *Bcl2*) and metastasis‐related genes (*MMP2* and *MMP9*) were evaluated using *β‐actin* as the endogenous reference gene. The primer sequences used in this study are listed in Table 1.

**TABLE 1:** Primer sequences used for real‐time PCR.

Gene	Primer sequence (5'→3')	Product size (bp)	Tm (°C)	Accession number
*β‐actin*	F: TCCTCCTGAGCGCAAGTAC R: CCTGCTTGCTGATCCACATCT	89	55	NM_001101.5
*Bax*	F: GAGCTGCAGAGGATGATTGC R: AAGTTGCCGTCAGAAAACATG	92	57	NM_138764.5
*Bcl2*	F: ATTGGGAAGTTTCAAATCAGC R:CAGTCTACTTCCTCTGTGATGTTG	150	55	NM_000657.3
*MMP2*	F: TTGACGGTAAGGACGGACTC R: CATACTTCACACGGACCACTTG	126	59	NM_004994.3
*MMP9*	F: GCACGACGTCTTCCAGTACC R:CAGGATGTCATAGGTCACGTAGC	124	60	NM_001302510.2

Each PCR reaction was performed in a final volume of 25 µL containing 12.5 µL SYBR Green PCR Master Mix, 1 µL cDNA template, forward and reverse primers (100 nM each), and nuclease‐free water. The amplification protocol consisted of an initial denaturation at 95°C for 5 min, followed by 35 cycles of denaturation at 95°C for 30 s, annealing at 54°C for 30 s, and extension at 72°C for 30 s.

Relative gene expression levels were calculated using the 2^−ΔΔCt^ method. All reactions were performed in triplicate, and the obtained data were analyzed using the comparative threshold cycle (Ct) method [24].

### 2.4 Flow Cytometry Analysis of Apoptosis

Apoptotic cell death was evaluated using an Annexin V‐FITC/Propidium Iodide (PI) Apoptosis Detection Kit (BD Biosciences, Franklin Lakes, NJ, USA). SKOV3 cells were seeded at a density of 1 × 10^4^ cells per well in 6‐well plates and treated with the respective 72 h IC_50_ concentration of each treatment, including free *O. basilicum* extract, blank niosomes, and *O. basilicum* extract‐loaded niosomes. Thus, each treatment was evaluated at its own treatment‐specific 72 h IC_50_ concentration rather than at a common concentration. After treatment, cells were washed twice with cold phosphate‐buffered saline (PBS, pH 7.4), detached using trypsin–EDTA, and resuspended in 1× binding buffer. Subsequently, the cells were stained with Annexin V‐FITC and propidium iodide (PI) and incubated for 10 min at room temperature in the dark, according to the manufacturer's instructions. Samples were analyzed using a flow cytometer (BD FACSCalibur, BD Biosciences, San Jose, CA, USA). Cell populations were classified as viable (Annexin V−/PI−), early apoptotic (Annexin V+/PI−), late apoptotic (Annexin V+/PI+), and necrotic (Annexin V−/PI+). Representative flow cytometry plots were generated using CellQuest and are presented in the Results section [25].

### 2.5 Statistical Analysis

All experiments were performed in triplicate, and the results are presented as mean ± standard deviation (SD). Statistical analyses were carried out using GraphPad Prism version 5.0 (GraphPad Software Inc., San Diego, CA, USA). Differences among experimental groups were analyzed using one‐way analysis of variance (ANOVA) followed by Tukey's multiple comparison post hoc test. A value of *p *< 0.05 was considered statistically significant [25].

## 3 Results and Discussion

### 3.1 Physicochemical Characterization of Nanoparticles

#### 3.1.1 Dynamic Light Scattering (DLS) Analysis

The hydrodynamic diameter of the prepared nanoparticles was determined by dynamic light scattering (DLS), and the results are presented in Figure 1. Blank niosomes exhibited an average particle size of approximately 200 nm, whereas *O. basilicum* extract‐loaded niosomes showed a mean diameter of approximately 400 nm. The increase in particle size following extract loading, together with the encapsulation efficiency and FTIR findings, supports the successful incorporation of the extract into the niosomal formulation.

**FIGURE 1:**
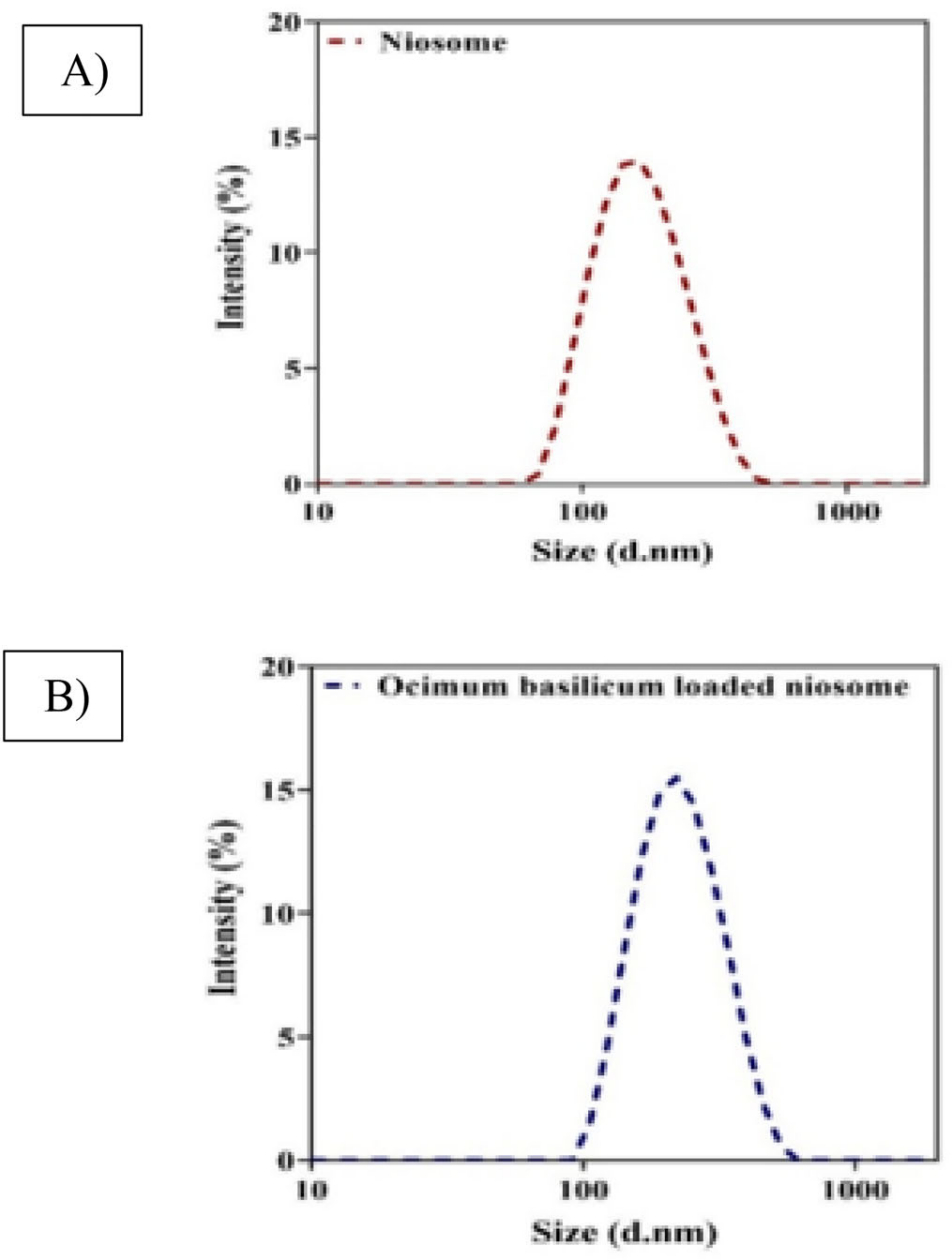
Dynamic light scattering (DLS) analysis showing the hydrodynamic diameter and particle size distribution of (A) free niosomes and (B) *O. basilicum* extract‐loaded niosomes. The increase in particle size following extract encapsulation indicates successful loading of the extract into the niosomal vesicles.

The particle size observed in the present study was within the nanoscale range and was considered compatible with the intended formulation characteristics. However, particle size alone does not establish superior delivery performance or biological efficacy, and its relevance should be considered together with the other physicochemical characteristics of the formulation. The observed increase in particle size after encapsulation is consistent with previous reports on plant extract‐loaded niosomes, in which loading of bioactive compounds resulted in larger vesicle diameters due to the incorporation of phytochemicals within the lipid bilayer [23, 25]. Thus, the DLS findings support successful formulation of the extract‐loaded niosomes but should not, by themselves, be interpreted as evidence of enhanced biological activity.

#### 3.1.2 Scanning Electron Microscopy (SEM) Analysis

Scanning electron microscopy (SEM) was performed to evaluate the morphology of the prepared nanoparticles. As shown in Figure 2, the *O. basilicum* extract‐loaded niosomes exhibited a predominantly spherical morphology with a relatively uniform size distribution and no obvious aggregation. The particle size estimated from the SEM micrographs ranged from 35.02 to 61.23 nm.

**FIGURE 2:**
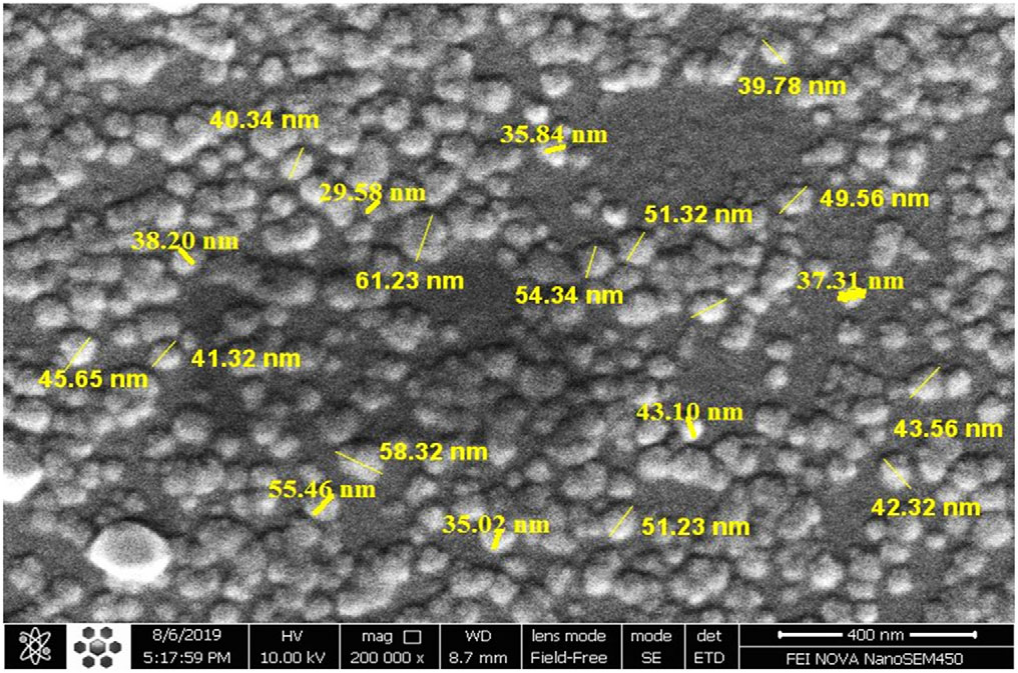
Scanning electron microscopy (SEM) image of *O. basilicum* extract‐loaded niosomes showing their spherical morphology and homogeneous particle distribution.

The predominantly spherical morphology provides supportive evidence of the structural characteristics of the formulation; however, cellular internalization was not directly evaluated in the present study and therefore cannot be inferred from SEM morphology alone. The smaller particle size obtained by SEM compared with DLS analysis is expected because SEM measures the diameter of dehydrated particles, whereas DLS determines the hydrodynamic diameter of particles dispersed in an aqueous medium. Similar differences between SEM and DLS measurements have been reported for other plant extract‐loaded niosomal formulations, confirming that these two techniques provide complementary information on nanoparticle characteristics.

#### 3.1.3 Fourier Transform Infrared (FTIR) Analysis

The Fourier‐transform infrared (FTIR) spectra of the prepared niosomes are presented in Figure 3. The spectra confirmed the presence of the characteristic functional groups of both the niosomal components and the encapsulated *O. basilicum* extract. In the extract‐loaded niosomes, absorption bands observed at 1475–1600 cm^−^^1^, 1700–1725 m^−^^1^, and 3600–3650 cm^−^^1^ were assigned to aromatic C═C stretching, carboxylate (RCOO^−^) stretching, and O─H stretching vibrations, respectively. In contrast, the blank niosomes exhibited characteristic absorption peaks at 1100 cm^−^^1^ (C─O stretching), 1736 cm^−^^1^ (C═O stretching), 2858–2920 cm^−^^1^ (C─H stretching), 3406 cm^−^^1^ (O─H stretching), and 1000–1292 cm^−^^1^ (C─N stretching), reflecting the chemical structure of the niosomal matrix.

**FIGURE 3:**
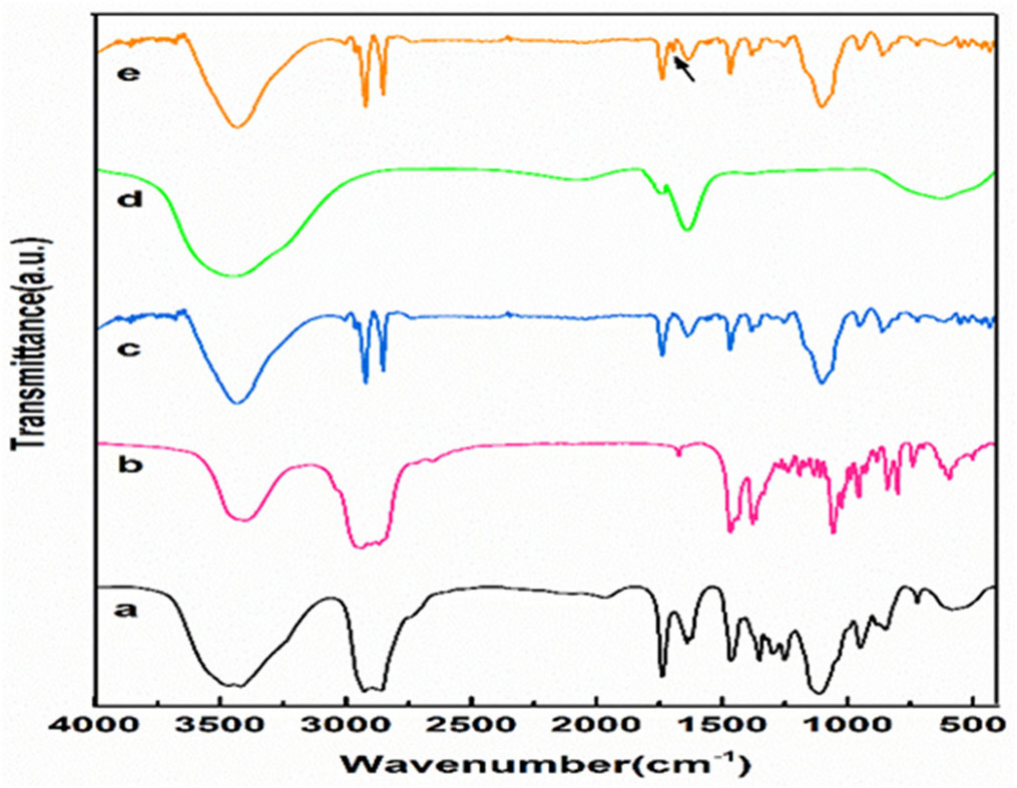
Fourier‐transform infrared (FTIR) spectra of (a) Span 60, (b) cholesterol, (c) free niosomes, (d) *O. basilicum* extract, and (e) *O. basilicum* extract‐loaded niosomes. The spectra were used to evaluate the characteristic functional groups and to assess potential interactions between the extract and the niosomal components following encapsulation.

Following encapsulation of the *O. basilicum* extract, the RCOO^−^ absorption band shifted from 1736 to 1699 cm^−^^1^, indicating interactions between the phytochemical constituents of the extract and the niosomal bilayer. No additional absorption bands or disappearance of the characteristic peaks was observed, suggesting that no major changes in the detectable functional groups occurred following encapsulation.

The observed peak shift and preservation of the characteristic FTIR bands provide evidence supporting the incorporation of the extract into the niosomal vesicles, rather than formation of new covalent bonds. Similar FTIR spectral changes have been reported for other plant extract‐loaded niosomal formulations, where slight shifts in absorption bands were attributed to hydrogen bonding and non‐covalent interactions between phytochemicals and the surfactant bilayer [26].

#### 3.1.4 In Vitro Drug Release Study

The in vitro release profile of *O. basilicum* extract from the prepared niosomes was evaluated in phosphate‐buffered saline (PBS, pH 7.4) at 37°C over a period of 72 h, and the results are presented in Figure 4. The free extract exhibited a rapid release profile and was almost completely released within the first 24 h. In contrast, the *O. basilicum* extract‐loaded niosomes showed a sustained release pattern, with approximately 55% of the encapsulated extract released after 24 h and approximately 60% after 72 h. The slower release observed for the niosomal formulation can be attributed to the presence of the lipid bilayer, which acts as a diffusion barrier and enables controlled release of the encapsulated phytochemicals.

**FIGURE 4:**
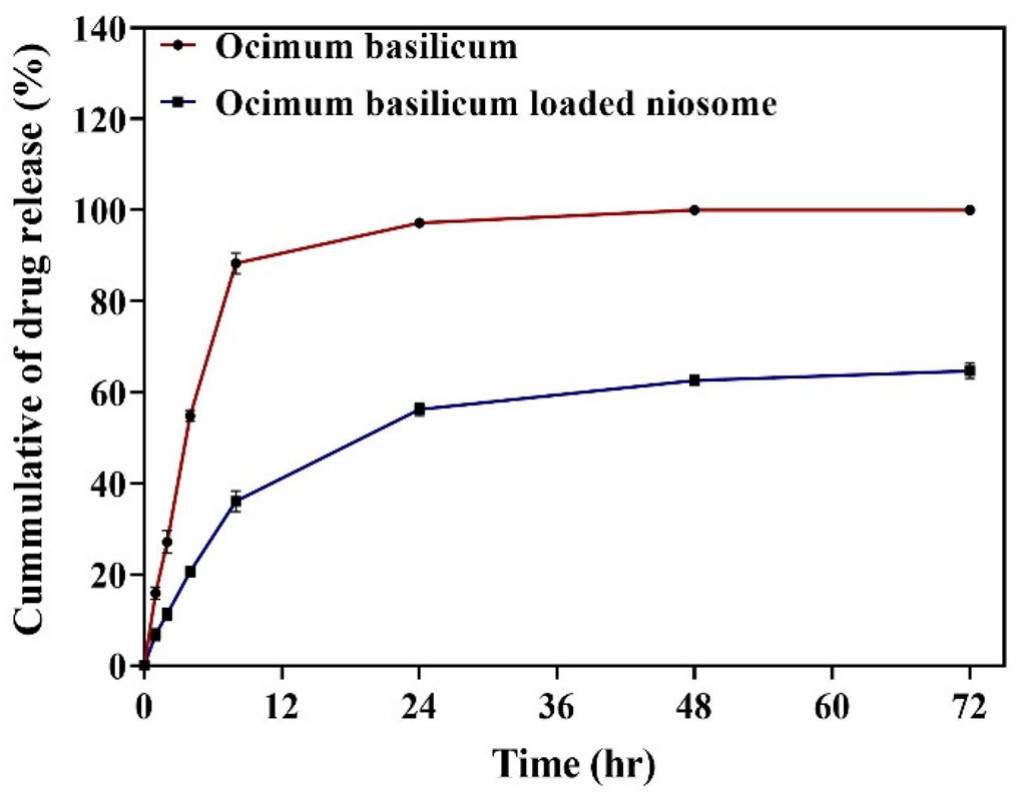
In vitro cumulative release profiles of free *O. basilicum* extract and *O. basilicum* extract‐loaded niosomes at pH 7.4 over 72 h, determined using the dialysis bag diffusion method. Data are presented as mean ± SD (*n* = 3).

To further elucidate the release mechanism, the experimental data were fitted to different kinetic models. Among the evaluated models, the Korsmeyer–Peppas model provided the best fit for the release profile. The calculated release exponent (*n *= 0.4253) indicated that the release of the encapsulated extract at pH 7.4 was predominantly governed by Fickian diffusion. These findings indicate that niosomal encapsulation provided a substantially slower release profile than the free extract, which may contribute to prolonged exposure to the encapsulated phytochemicals. However, the present release study does not establish that the sustained‐release behavior directly accounts for the enhanced cytotoxic effects observed in SKOV3 cells. Similar sustained‐release behavior has been reported for other plant extract‐loaded niosomal delivery systems.

Rapid advances in nanotechnology have created new opportunities for improving the delivery and therapeutic efficacy of natural bioactive compounds in cancer treatment. Among the various nanocarrier systems, niosomes have attracted considerable attention because of their potential to improve drug stability, facilitate controlled release, and enhance the biological activity of encapsulated phytochemicals [26, 27]. Recent studies have further highlighted the potential of nanotechnology‐based delivery systems for overcoming poor bioavailability and improving the therapeutic efficacy of natural anticancer agents [28, 29].

In the present study, *O. basilicum* extract‐loaded niosomes were successfully prepared and characterized using DLS, SEM, FTIR spectroscopy, encapsulation efficiency, and in vitro drug‐release analyses. The physicochemical characterization confirmed the successful incorporation of the extract into niosomal vesicles with the observed size distribution and sustained‐release behavior. The slower release profile observed for the niosomal formulation compared with the free extract is consistent with the barrier function of the lipid bilayer, which may prolong the diffusion of encapsulated phytochemicals and thereby contribute to prolonged biological activity [19, 20].

### 3.2 Time‐Dependent Cytotoxicity of *O. basilicum* Extract‐Loaded Niosomes

The cytotoxic effects of free *O. basilicum* extract, blank niosomes, and *O. basilicum* extract‐loaded niosomes on SKOV3 cells were evaluated using the MTT assay after 24, 48, and 72 h of treatment (Figure 5). A concentration‐ and time‐dependent reduction in cell viability was observed in all treatment groups. However, the extract‐loaded niosomes exhibited significantly greater cytotoxic activity than both the free extract and blank niosomes at all evaluated time points (*p *< 0.001). The IC_50_ values of the extract‐loaded niosomes decreased from 676.35 µg/mL at 24 h to 524.87 µg/mL at 48 h and 398.24 µg/mL at 72 h, indicating increased cytotoxic potency with prolonged exposure. In comparison, the free extract and blank niosomes showed considerably higher IC_50_ values throughout the experimental period, indicating lower cytotoxic potency.

**FIGURE 5:**
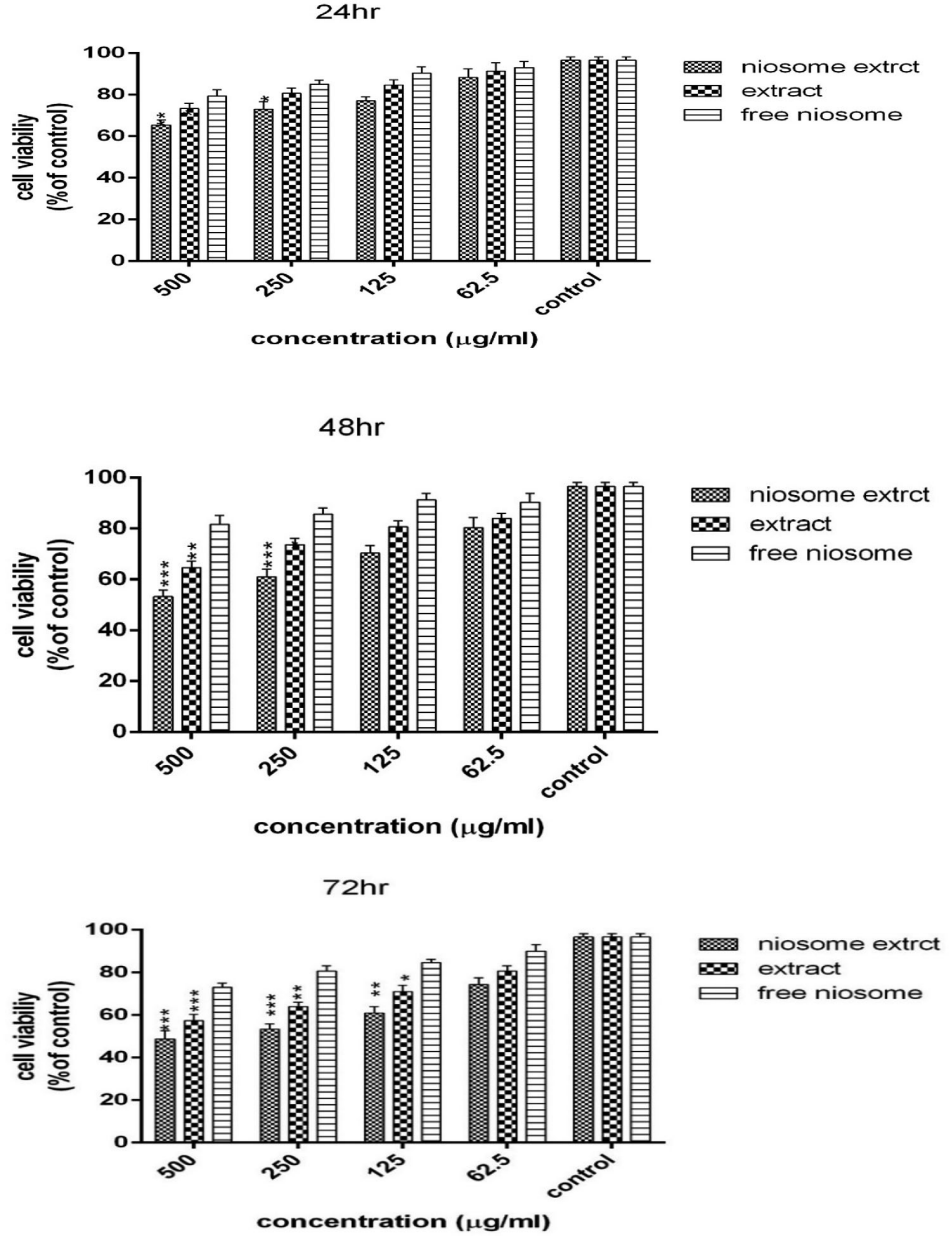
Effects of free *O. basilicum* extract, blank niosomes, and *O. basilicum* extract‐loaded niosomes on the viability of SKOV3 cells after 24, 48, and 72 h, as determined by the MTT assay. Data are presented as mean ± SD (*n* = 3). Statistical significance was determined by one‐way ANOVA followed by Tukey's post hoc test. *p* < 0.05, *p* < 0.01, and *p* < 0.001 versus the untreated control group.

A notable finding of the present study was the consistently greater cytotoxic response of the extract‐loaded niosomes compared with the free extract across the three exposure periods, indicating that incorporation into the niosomal system was associated with enhanced cytotoxic potency in the SKOV3 model. The greater cytotoxic activity observed for the extract‐loaded niosomes may be related to the physicochemical characteristics of the formulation and its sustained‐release behavior. However, because cellular uptake and intracellular retention were not directly evaluated in the present study, these mechanisms cannot be confirmed experimentally and should be considered potential explanations. Similar enhancement of anticancer activity following niosomal encapsulation has been reported for other plant‐derived bioactive compounds, including flavonoids and phenolic compounds, and has been associated with improved bioavailability and controlled drug release [20, 21, 22, 28, 29]. Thus, the present findings support an association between niosomal encapsulation and enhanced cytotoxic potency of the *O. basilicum* extract in SKOV3 cells, although the specific contribution of cellular uptake, intracellular retention, and release kinetics remains to be determined.

### 3.3 Gene Expression Analysis

The expression levels of the apoptosis‐related genes (*Bax* and *Bcl2*) and the metastasis‐associated genes (*MMP2* and *MMP9*) were quantified by real‐time PCR in SKOV3 cells treated with free *O. basilicum* extract, blank niosomes, and *O. basilicum* extract‐loaded niosomes for 24, 48, and 72 h. The results are presented in Figure 6.

**FIGURE 6:**
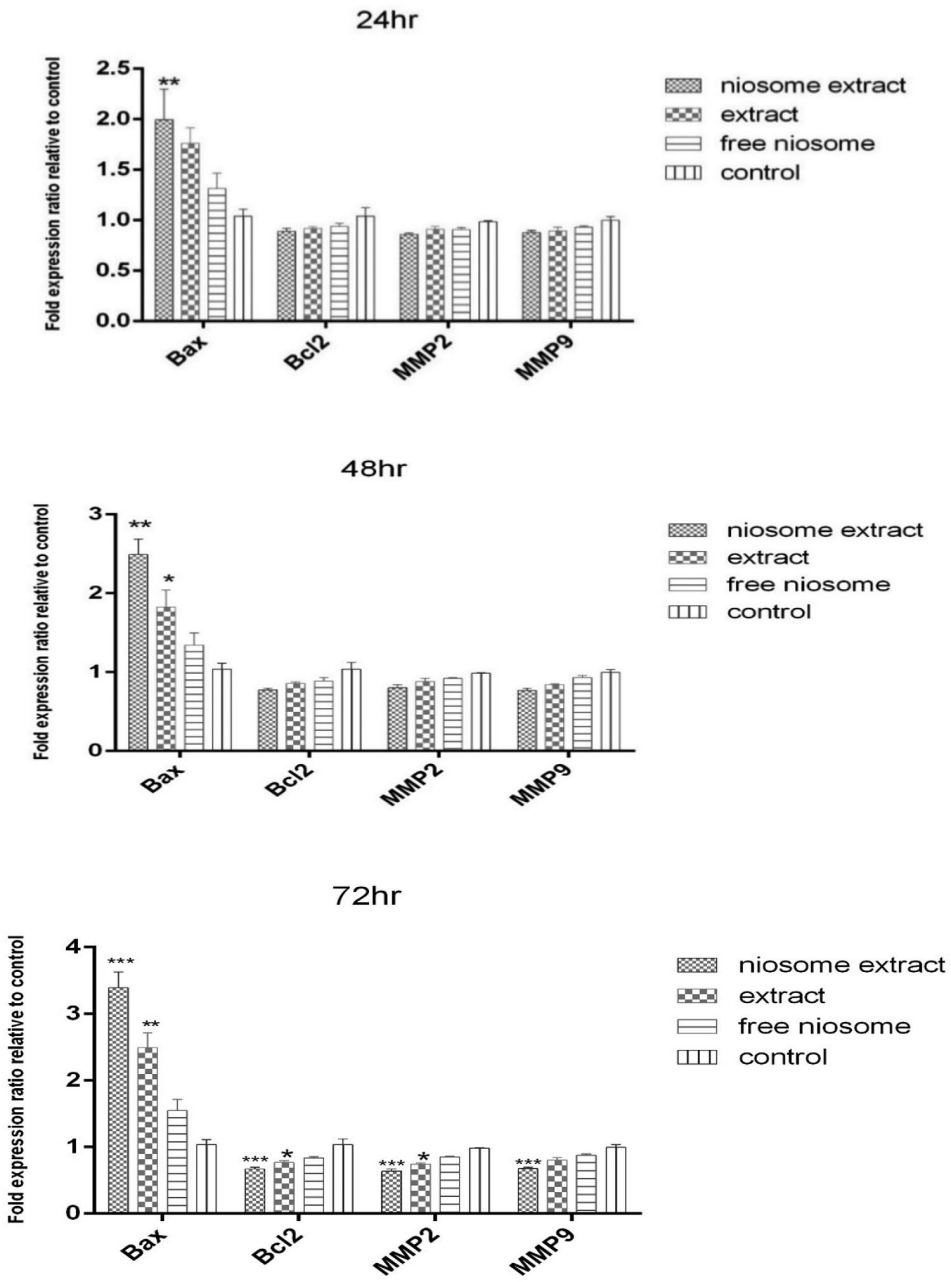
Relative gene expression levels of *Bax*, *Bcl2*, *MMP2*, and *MMP9* in SKOV3 cells following treatment with free *O. basilicum* extract, blank niosomes, and *O. basilicum* extract‐loaded niosomes for 24, 48, and 72 h, as determined by real‐time quantitative PCR (RT‐qPCR). Gene expression levels were normalized to ***β‐actin***. Data are presented as mean ± SD of three independent experiments. Statistical significance was determined by one‐way ANOVA followed by Tukey's post hoc test. *p* < 0.05, *p* < 0.01, and *p* < 0.001 versus the untreated control group.

After 24 h of treatment, the extract‐loaded niosomes significantly increased *Bax* expression by approximately 1.91‐fold (*p* < 0.01), whereas no statistically significant changes were observed in the expression of *Bcl2*, *MMP2*, or *MMP9*. Neither the free extract nor the blank niosomes produced significant changes in the evaluated genes at this time point. Following 48 h of treatment, *Bax* expression was further increased in cells treated with the extract‐loaded niosomes (approximately 2.43‐fold, *p* < 0.01), while the free extract induced a moderate increase in *Bax* expression (approximately 1.78‐fold, *p* < 0.05). The expression levels of *Bcl2*, *MMP2*, and *MMP9* remained unchanged at this stage.

The most pronounced transcriptional changes were observed after 72 h of treatment. At this time point, the extract‐loaded niosomes significantly upregulated *Bax* expression (approximately 3.46‐fold, *p* < 0.001) and significantly reduced the expression of *Bcl2* (approximately 0.67‐fold, *p* < 0.001), *MMP2* (approximately 0.63‐fold, *p* < 0.001), and *MMP9* (approximately 0.64‐fold, *p* < 0.001). Treatment with the free extract also increased *Bax* expression (approximately 2.47‐fold, *p* < 0.01), but produced comparatively smaller effects on the other evaluated genes.

The progressive increase in *Bax* expression together with the reduction in *Bcl2* at 72 h resulted in a transcriptional profile consistent with enhanced pro‐apoptotic signaling. Similarly, the reduced expression of *MMP2* and *MMP9* suggests modulation of genes associated with extracellular matrix remodeling and migration‐related processes. However, these findings represent transcriptional changes and should not be interpreted as definitive evidence of activation of a specific apoptotic pathway or inhibition of metastasis, particularly in the absence of protein‐level validation.

The observed molecular changes are broadly consistent with previous reports on the biological activity of *O. basilicum* and its phytochemical constituents. Rauf et al. reported that phenolic constituents of basil can modulate apoptosis‐related processes by increasing the expression of pro‐apoptotic mediators while suppressing anti‐apoptotic signaling [30]. Torres et al. also reported cytotoxic activity of aqueous *O. basilicum* extract against MCF‐7 breast cancer cells, although the reported IC_50_ values were higher than those observed for the niosomal formulation in the present study [31]. These findings provide contextual support for the biological activity of *O. basilicum*, while the present results further indicate that incorporation of the extract into niosomes is associated with a greater magnitude of transcriptional responses in SKOV3 cells.

Recent studies have likewise reported that nanoformulation of phytochemicals can enhance their biological efficacy through controlled release and improved bioavailability [26, 28, 29, 32].

Importantly, the transcriptional findings should be considered together with the flow cytometry results presented in the following section. The increased apoptotic cell population observed following treatment with extract‐loaded niosomes provides complementary evidence of apoptotic cell death and is consistent with the observed changes in *Bax* and *Bcl2* expression. Nevertheless, because the present molecular analysis was performed at the mRNA level, the involvement of the corresponding proteins and downstream signaling events cannot be established definitively. Protein‐level analyses of BAX, BCL2, and other apoptosis‐related markers would therefore be necessary to determine whether the observed transcriptional alterations translate into corresponding changes at the protein level.

### 3.4 Flow Cytometry Analysis of Apoptosis

Flow cytometry analysis was performed to quantify apoptotic cell death in SKOV3 cells following treatment with the IC_50_ concentrations of free *O. basilicum* extract, blank niosomes, and *O. basilicum* extract‐loaded niosomes for 24, 48, and 72 h. The representative flow cytometry plots and quantitative analysis are presented in Figures 7 and 8.

**FIGURE 7:**
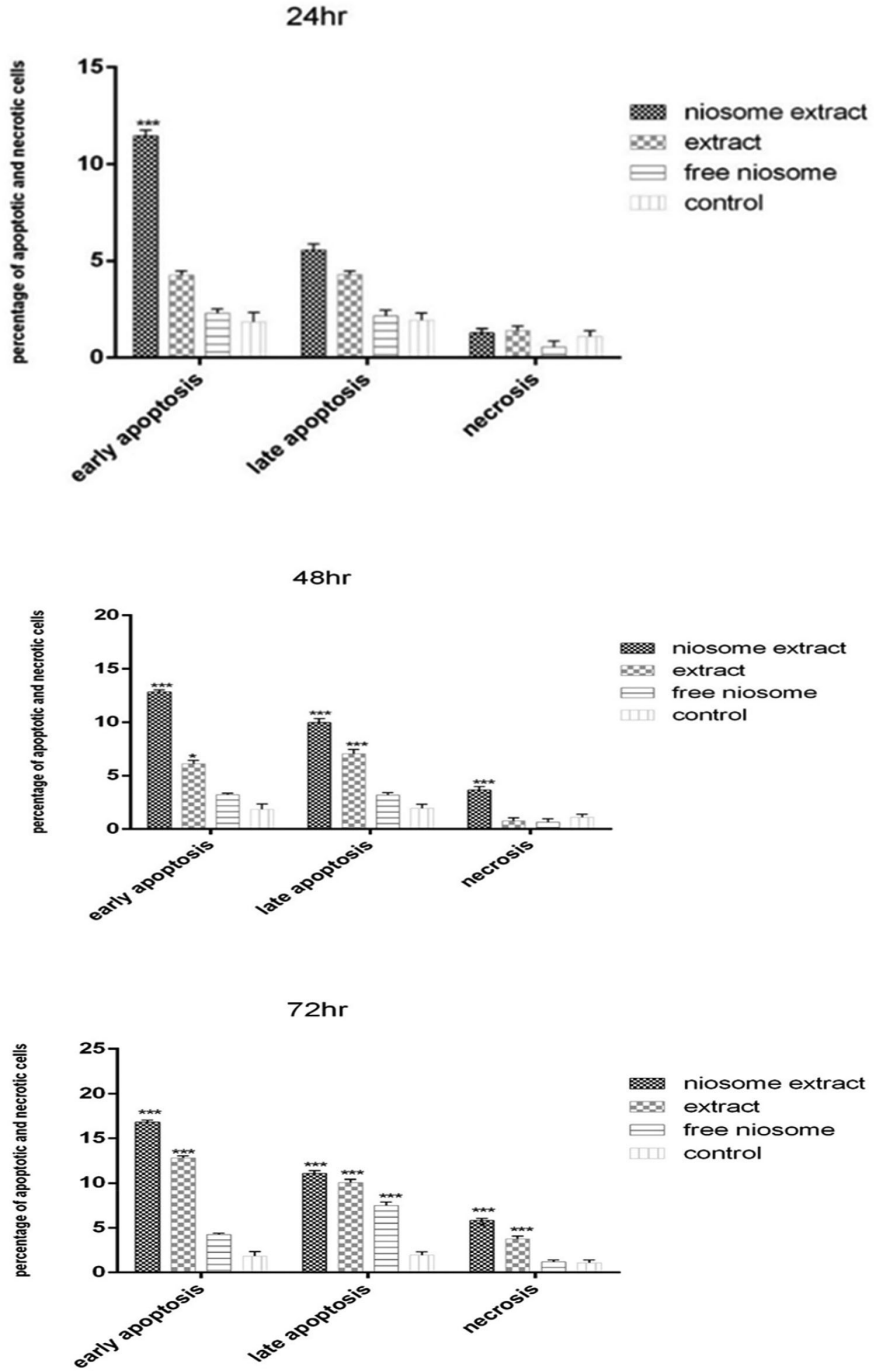
Flow cytometric analysis of apoptosis in SKOV3 cells following treatment with free *O. basilicum* extract, free niosomes, and *O. basilicum* extract‐loaded niosomes for 24, 48, and 72 h using Annexin V‐FITC/propidium iodide (PI) staining. Representative flow cytometry dot plots and quantitative analysis of viable, early apoptotic, late apoptotic, and necrotic cell populations are shown. Data are presented as mean ± SD of three independent experiments. Statistical significance was determined by one‐way ANOVA followed by Tukey's post hoc test. *p* < 0.05, *p* < 0.01, and *p* < 0.001 versus the untreated control group.

**FIGURE 8:**
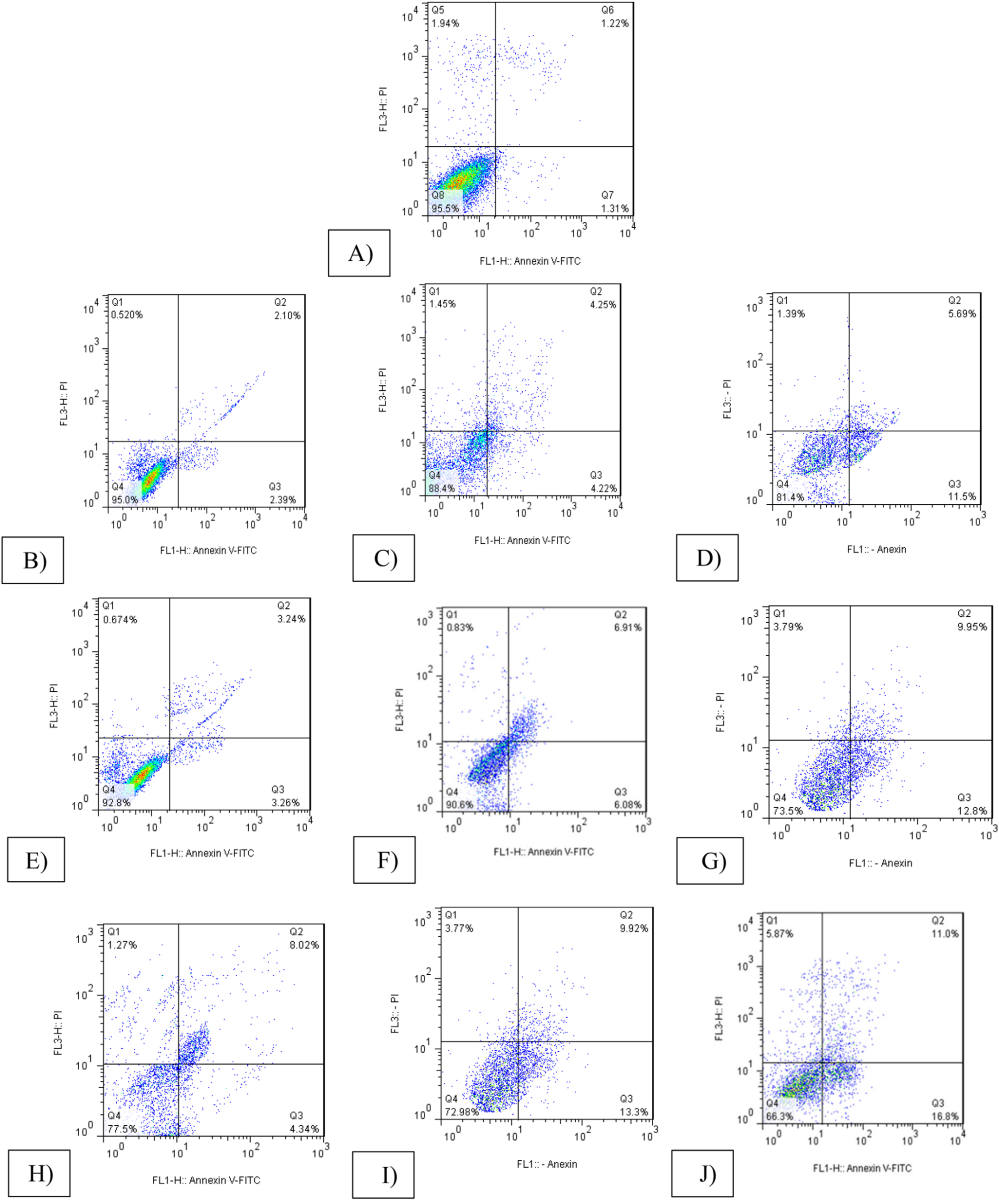
Flow cytometric analysis of apoptosis induction in SKOV3 cells following treatment with blank niosomes, free *O. basilicum* extract, and *O. basilicum* extract‐loaded niosomes for 24, 48, and 72 h. Each treatment was applied at its respective 72 h IC_50_ concentration (A) Untreated control at 24 h; (B) blank niosomes at 24 h; (C) free extract at 24 h; (D) extract‐loaded niosomes at 24 h; (E) blank niosomes at 48 h; (F) free extract at 48 h; (G) extract‐loaded niosomes at 48 h; (H) blank niosomes at 72 h; (I) free extract at 72 h; and (J) extract‐loaded niosomes at 72 h. The percentage of cells in viable, early apoptotic, late apoptotic, and necrotic populations was determined by flow cytometry based on Annexin V‐FITC/PI staining.

After 24 h of treatment, *O. basilicum* extract‐loaded niosomes induced a total apoptotic population of 17.19%, comprising 11.50% early apoptotic and 5.69% late apoptotic cells. The corresponding total apoptotic populations for the free extract and blank niosomes were 8.47% and 4.94%, respectively.

Following 48 h of treatment, the percentage of apoptotic cells increased to 22.75% in the extract‐loaded niosome group, including 12.80% early apoptotic and 9.95% late apoptotic cells. In comparison, the free extract induced 12.99% total apoptosis, whereas the blank niosomes resulted in 4.94% apoptosis.

The highest apoptotic response was observed after 72 h of treatment with the extract‐loaded niosomes, which produced 27.80% total apoptosis, consisting of 16.80% early apoptotic and 11.00% late apoptotic cells. In comparison, treatment with the free extract resulted in 22.75% apoptosis, whereas the blank niosomes induced 12.36% apoptosis.

The progressive increase in apoptotic cell populations following treatment with the extract‐loaded niosomes was accompanied by time‐dependent transcriptional changes, particularly increased *Bax* expression and reduced *Bcl2* expression at 72 h. These findings provide complementary evidence that the observed transcriptional changes occurred alongside increased apoptotic cell death. However, the present data do not establish a direct causal relationship between these transcriptional alterations and apoptosis or confirm activation of a specific apoptotic pathway. Because the molecular analysis was performed at the mRNA level, corresponding changes at the protein level cannot be established. Protein‐expression analyses of apoptosis‐related markers would therefore be valuable for further elucidating the molecular basis of the observed apoptotic response.

The observed apoptotic response provides functional evidence of increased apoptotic cell death following treatment with the *O. basilicum* extract‐loaded niosomes. Nevertheless, the findings should be interpreted within the scope of the experimental model, and additional protein‐level analyses and in vivo studies are required to determine whether the observed molecular and cellular effects translate into broader antitumor activity.

## 4 Novelty and Contribution of the Present Findings

A key finding of the present study was that incorporation of *O. basilicum* extract into niosomes was associated with greater cytotoxic potency in SKOV3 cells than the free extract, together with a time‐dependent increase in apoptotic cell populations. These cellular effects were accompanied by coordinated transcriptional changes, including increased *Bax* and reduced *Bcl2* expression at 72 h. Although these individual molecular markers and their association with *O. basilicum* bioactivity have been reported previously, the present findings demonstrate that the specific extract‐loaded niosomal formulation produced a consistent pattern of enhanced cytotoxicity and apoptotic response in the SKOV3 model. Thus, the principal contribution of the present study is not the identification of previously unknown molecular markers, but the experimental demonstration of the biological response associated with this specific niosomal formulation and its relationship with the observed physicochemical and release characteristics.

## 5 Conclusion

This study demonstrated that *O. basilicum* extract‐loaded niosomes reduced the viability of SKOV3 ovarian cancer cells more effectively than the free extract and induced a time‐dependent increase in apoptotic cell death. Treatment with the niosomal formulation resulted in significant modulation of apoptosis‐related genes (*Bax* and *Bcl2*) and changes in the expression of migration‐associated genes (*MMP2* and *MMP9*), particularly after 72 h of exposure. Together with the flow cytometry findings, these results support the pro‐apoptotic potential of the extract‐loaded niosomal formulation in the present in vitro model. However, the gene‐expression findings represent transcript‐level changes and should not be interpreted as definitive evidence of specific molecular pathway activation or anti‐metastatic activity.

These findings support further investigation of *O. basilicum* extract‐loaded niosomes as a potential nanocarrier system for plant‐derived anticancer compounds. The principal contribution of the present study is the integrated characterization of this specific extract‐loaded niosomal formulation and the demonstration of its cytotoxic and pro‐apoptotic potential in SKOV3 cells. However, protein‐level validation, additional functional migration and invasion assays, evaluation in additional cancer and non‐cancer cell models, and in vivo studies are required to determine whether the observed molecular and cellular effects can be reproduced in broader biological systems and ultimately translate into therapeutic benefit.

## 6 Limitations

The present study has several limitations. First, the biological effects of the formulation were evaluated in a single ovarian cancer cell line, which may limit the generalizability of the findings to other ovarian cancer subtypes and to non‐cancerous cells. Second, although the physicochemical characteristics, encapsulation efficiency, and in vitro release profile of the formulation were determined, long‐term formulation stability was not evaluated. Therefore, the stability of the formulation during prolonged storage and its potential influence on biological activity remain to be established.

Third, the molecular analyses were performed at the transcript level, and protein‐level validation, including assessment of *Bax*, *Bcl2*, and other apoptosis‐related proteins, was not performed. Therefore, the observed changes in gene expression should not be interpreted as definitive evidence of corresponding changes in protein activity or signaling pathway activation. In particular, the observed increase in *Bax* and decrease in *Bcl2* transcripts provide supportive molecular evidence but do not establish activation of a specific apoptotic signaling pathway.

Fourth, cellular uptake and intracellular accumulation of the encapsulated extract were not directly assessed. Consequently, the mechanisms underlying the greater cytotoxic activity of the extract‐loaded niosomes, including enhanced cellular uptake, intracellular retention, or sustained intracellular exposure, cannot be distinguished on the basis of the present data.

Fifth, although *MMP2* and *MMP9* expression was evaluated, functional migration and invasion assays were not performed; therefore, the present findings do not establish an anti‐metastatic effect. The observed changes in these transcripts should instead be considered modulation of migration‐ and extracellular‐matrix‐associated gene expression, requiring functional validation before any conclusion regarding altered migratory or invasive behavior can be drawn.

Finally, the therapeutic efficacy and safety of the formulation remain to be validated in appropriate in vivo models. The present findings are therefore limited to an in vitro experimental model and should not be extrapolated directly to therapeutic efficacy in vivo. Future studies should incorporate protein‐level analyses, complementary functional assays, multiple relevant cell models, pharmacokinetic and biodistribution assessments where appropriate, and in vivo efficacy and safety studies to further define the biological and translational significance of the formulation.

## Author Contributions

**Zahra Mousavi**: conceptualization, investigation, data curation, visualization, writing – original draft. **Mahsa Kavousi**: conceptualization, methodology, validation, formal analysis, supervision, writing – review and editing. **Mahnaz Farahmand**: supervision, writing – review and editing. All authors read and approved the final manuscript.

## Funding

The authors declare that no funding was received from any organization or agency in support of this research.

## Use of Generative AI and AI‐Assisted Technologies in the Writing Process

During the preparation of this manuscript, the authors did not use an AI‐assisted language tool to generate or manipulate experimental data, perform statistical analyses, interpret scientific findings, or make scientific decisions. All scientific content, interpretations, conclusions, and the final version of the manuscript were reviewed, verified, and approved by the authors, who take full responsibility for the content of the manuscript.

## Ethics Statement

The present study investigated the effects of Ocimum extract using the SKOV3 cell line under in vitro conditions. No human participants or animals were involved in this research. Therefore, ethical approval and an ethics committee code were not required in accordance with institutional and international guidelines.

## Conflicts of Interest

The authors declare no conflicts of interest.

## Data Availability

The materials used in this study are available from the corresponding author upon request.
